# Epidemiology of age-related macular degeneration among elderly in geriatric homes, East Cairo, Egypt

**DOI:** 10.1186/s12889-025-23680-6

**Published:** 2025-07-18

**Authors:** Dina Hussien Edarous, Hoda I. Fahim, Mohamed Momen, Ashraf Abd El-Salam Shaat, Ayat F. Manzour

**Affiliations:** 1 Department of Community, Environmental, and Occupational Medicine, Faculty of Medicine, Ain Shams University, Cairo, Egypt; 2 Department of Ophthalmology - Faculty of medicine, Ain Shams University, Cairo, Egypt

**Keywords:** Age-related macular degeneration, Macular dystrophies, Visual impairment, Elderly, Geographic atrophy, Drusen, Falls, Functional limitation

## Abstract

**Background:**

Visual impairment is one of the main causes of years lived with disability among adults 65 and older worldwide, according to the Global Burden of Disease Study (GBD). Both ageing and visual impairment are linked to an increased risk of falling, accounting for 40% of all injury-related deaths among older adults.

**Objective:**

To determine the prevalence of AMD among a sample of elderly residing in geriatric homes, identify associated factors of AMD, and to find out the effect of AMD on fall frequency in the studied group.

**Methods:**

A cross-sectional study was conducted on 283 elderly people in thirty geriatric homes in East Cairo. Data was collected using a structured interview questionnaire, and fundus examination for diagnosis.

**Results:**

The average age of study participants was 73.18 ± 7.09 (60–96) years. AMD was present in 12.7% of the patients. Early AMD was present in 58.4% of AMD patients, whereas 8.3% and 33.3% of the participants had late dry and late wet AMD, respectively. Bilateral AMD was found in 38.9%, compared to 61.1% who had unilateral AMD. Compared to other age groups, the prevalence of AMD was significantly higher in people aging 75 + years. About 47.2% of participants diagnosed with AMD were current smokers. Out of the participants diagnosed with AMD, 72.2% were diabetics and 91.7% were hypertensives. About 52.8% and 36.1% of AMD participants had moderate and high risk of fall according to the Morse Fall scale, respectively, while 52.7% and 30.6% of them had moderate and severe ADL impairment and scored below average in the IADL questionnaire compared to their normal peers.

**Conclusion:**

The prevalence of AMD in Egypt is higher than previously documented. The most common form of AMD was early, and the late wet variety was second. Important non-modifiable risk factors for AMD include advancing age, being a woman, and having a positive family history of the disease. Additionally, smoking, diabetes, and hypertension are important risk factors for AMD. Vitamin A consumption appears to offer some protection against AMD. Compared to older adults without AMD, AMD patients had a higher risk of falls and more impairment in ADL and IADL.

**Supplementary Information:**

The online version contains supplementary material available at 10.1186/s12889-025-23680-6.

## Introduction

One of humanity’s greatest accomplishments is the increase in longevity. Better nutrition, sanitation, health care, education, and economic prosperity all contribute to longer lifespans. But growing older is like a two-sided medal [1].

Visual impairment (VI) is regarded as one of the most serious medical conditions. There were an estimated 253 million visually impaired people globally in 2017 [2]. At least 2.2 billion individuals worldwide had VI as of 2020. However, the exclusion of refractive error as a cause of VI suggests that the true global magnitude of VI is higher [3]. By the time they are 60, about one in three older people suffers from a vision-impairing eye condition [4]. The most common causes of visual affection globally, other than refractive errors, are glaucoma, age-related macular degeneration (AMD), diabetic retinopathy, and cataracts [5].

AMD is a progressive degenerative disease of the macula. One of its defining features is loss of central vision [6]. AMD has been classified into early and late forms by the Age-Related Eye Disease Study (AREDS), Beckman, and Rotterdam classifications. Two more classifications for late AMD are neovascular (wet, exudative form) and geographical atrophy (dry, nonexudative form) [7].

The precise causes of AMD are yet unknown, although they most likely involve a complex interplay of genetic, metabolic, and environmental variables [8]. Risk factors of AMD are numerous including age, gender, elevated body mass index (BMI), family history of the disease, smoking, alcohol consumption, and chronic illnesses like diabetes mellitus and hypertension [7].

AMD-related visually impaired seniors arrive at retirement homes roughly three years before their counterparts who have normal vision [9]. For everyday activities and household chores including grooming, cooking, feeding themselves, using phones, and handling family finances, AMD patients are more dependent on others, especially those who need to walk inside their homes and climb stairs [9]. 

A higher risk of falling is linked to both ageing and impairment of vision. Two-thirds of AMD patients showed poorer foot placement, slower gait speed, visual reaction times, and trouble stepping on low contrast targets when compared to controls with similar ages [10]. Compared to older adults without AMD, AMD patients are four times more likely to suffer from hip fractures and twice as likely to fall [11].

Though the precise etiology of AMD remains unclear, oxidative stress has been found to be a significant contributing factor. Conversely, it was proposed that increasing dietary consumption of carotenoids would lower the risk and slow AMD progress [12].

Significant changes in risk profiles and treatment options throughout time may have affected the likelihood and course of the disease. Furthermore, there is limited data on the prevalence of AMD in Egypt; instead, the majority of studies concentrate on treatment options. Therefore, the current study aims to determine the prevalence of AMD. It also aims to identify the characteristics related to AMD and to determine the association between fall occurrences in older adults and AMD.

## Methodology

A cross-sectional study was conducted to meet the objectives of the research. The study was carried out in all geriatric homes located in East Cairo, which totaled thirty homes. Of these, nine were privately operated and charged fees, while the remaining homes were funded by non-governmental organizations (NGOs), offering services for free or at minimal cost to those unable to pay. Each geriatric home housed an average of 15 to 18 elderly residents. There was an 84% response rate after the administration of five residences declined to participate. The study included all seniors of participating residences who provided their consent.

- The study population included elderly individuals aged 60 years and above. Data collection was conducted over a six-month period. Individuals were excluded if they:
  - ◦ Had dementia or mild cognitive impairment.
  - ◦ Were diagnosed (by history or examination) with ocular conditions, including:
    - ▪ Central corneal opacities
    - ▪ Advanced glaucoma
    - ▪ Advanced cataract
    - ▪ Extensive retinal diseases such as diabetic macular edema (DME)
- These exclusions were made because:
  - ◦ Conditions like central corneal opacity or advanced cataract hinder fundus examination. 
  - ◦ Advanced glaucoma due to optic nerve affection and DME affects central and peripheral vision, which may mimic age-related macular degeneration (AMD).

The required sample size was calculated using the PASS software (version 15), assuming a prevalence of AMD among the elderly population at 20.5% [13], with a 95% confidence level. Based on this, a minimum of 280 elderly participants was needed. Initially, 406 individuals were examined; however, only 283 met the inclusion criteria and were retained for the final analysis, while 123 were excluded based on the exclusion criteria (Supplementary Fig. S1).

### Study tools

Data collection was done using:

*Mini mental state examination (MMSE)* To exclude out cases of mild cognitive impairment or dementia. It has a maximum score of 30 points and assesses a few everyday mental skills. Mild, moderate, and severe dementia are indicated by scores between 20 and 24, 13 and 20, and less than 12, respectively. Participants who scored 25 or higher were included [14].

*Structured interview* questionnaire includes the following sections:

- Section I: Sociodemographic features of participants, e.g., (age, sex, education & previous occupation) [15].
- Section II: History of eye surgeries or any ophthalmological condition affecting vision (glaucoma, cataract, and severe retinal diseases) for exclusion.
- Section III: History of special habits of medical importance e.g., smoking, and alcohol [15].
- Section IV: Medical history of chronic diseases as hypertension (HTN), Diabetes mellitus (DM), hyperlipidaemia, and inner ear disease [15].
- Section V: Medications History, Dietary Supplements (type if any) and Medications History (especially Anticoagulants). Medication history was confirmed by care givers in the geriatric home and revised by researcher [15].
- Section VI: History of falls in the past year.
- Section VII: Morse Fall Scale (MFS) was used to determine the probability of falling. Three danger levels are indicated by the final score: a fall risk of less than 25, a fall risk of 25 to 45, and a fall risk of more than 45 [16].
- Section VIII: Family history of AMD.
- Section IX: Questionnaires on Instrumental Activities of Daily Living (IADL) and Activities of Daily Living (ADL). ADL investigates basic self-care and basic physical needs management activities. The primary areas of ADL include clothing, eating, bathing, walking, using the restroom, and maintaining one’s continence. A score of 6 indicates full function, a score of 4 indicates moderate impairment, while a score of 2 or fewer points to severe impairment. Whereas IADL encompasses activities required for self-sufficient functioning in the community. These include using phones, cooking, cleaning, traveling, buying groceries, doing laundry, taking prescribed medications, and handling finances. When all eight categories of function are applied to women, the total ranges from 0 (which indicates dependency) to 8 (which indicates high function as independence). While for men, preparing food, cleaning the house, and doing laundry are not considered. Men’s scores range from 0 to 5. Validated Arabic questionnaires were used [15].

*Anthropometric measurements*: A digital scale and measuring tape were used to determine height and weight, then compute the body mass index (BMI) weight/height “m2”.

### Fundus examination

- § Fundus examination was done by a specialised ophthalmologist for all participants to exclude other eye diseases, confirm the diagnosis of AMD and determine the type of AMD.
- § Patients were examined following pupillary dilation using tropicamide 1% eye drops “1% Mydriacyl” (Alcon-Couvreur, Puurs, Belgium) instilled 20–30 min prior to examination with an indirect binocular ophthalmoscope (Keeler Ltd, Halma, Berksire, UK) and a double aspheric 20 diopter Volk lens (Volk Optical Inc., Halma, Ohio, USA). Patients sat 25 cm from the indirect ophthalmoscope, and the lens was handheld in front of the examined eye, and then the patient was asked to look in cardinal gazes for fundus examination.

### Operational definitions [7]

*Early-stage AMD*: Presence of yellowish pigmentation in the centre of the retina called soft drusen.

*Late-stage AMD*: it’s either a wet type characterised by neovascularization or a dry type with geographic atrophy.

*Scotoma*: A spot in the visual field in which vision is absent or deficient.

*Metamorphopsia*: A visual problem that causes linear objects appear as curvy or rounded.

*Drusen*: Extracellular deposit of cellular debris from metabolic process, located beneath both Bruch’s membrane and the retinal epithelium (RPE). They consist of fat, amyloid, and complement factor.

### Data management

The Statistical Package for Social Sciences (SPSS) was used to collect, code, and analyze data. The data was processed according to its type, using mean ± standard deviation for quantitative data, while number and percentage were used for qualitative data. Independent samples t-test was used to compare quantitative variables between groups, and chi-square test was used to analyze the relationship between two qualitative variables. Binary logistic regression was used to forecast the relationship between independent variables and AMD. Statistical significance was defined as a *p*-value of less than 0.05.

## Results

About 406 elders were interviewed in the first place, and 123 were excluded based on the exclusion criteria (Fig. S1). The final analysis included 283 participants, with a mean age (± SD) of 73.18 (± 7.09) years, 36.80% of whom were older than 75, and a female predominance (52.7%). Of the participants, 65.7% had completed high school or more, while 34.3% had less than a high school education. Of the participants, 41% were classified as blue code and 25.10% were classified as white in terms of occupation; only 3.2% of the participants had a family history of AMD, and 48.4% were underweight and 7.4% were overweight (Table 1).

**Table 1:** Characteristics of study participants

Variable	*N*	%
Age groups		
60–64	13	4.6
65–69	85	30
70–74	81	28.6
≥ 75	104	36.8
Mean ± SD (min. – max.)	73.18 ± 7.09 (60–96)	
Gender		
-Male	134	47.3
-Female	149	52.7
Educational level		
-Illiterate/can read or write	66	23.3
-Primary/Middle school	31	11
-High school/Diploma	119	42
-University/high	67	23.7
Previous occupation^a^		
-Blue Code	116	41.0
-White Code	71	25.1
-Pink Code	96	33.9
Positive Family history of AMD	9	3.2
Weight (kg)	65.67 ± 9.47 (45–95)	
Height (cm)	165.67 ± 7.11 (149–180)	
Body mass index (BMI)	23.89 ± 3.15(17.8–33.59)	
BMI Categories^b^		
Under weight (< 23)	137	48.4
Normal weight (23–30)	125	44.2
Overweight(> 31)	21	7.4

^a^Collar classification of occupations: blue code: labor jobs, White code: jobs in office settings, Pink code: housewives. (Rozell et al., [17]); ^b^(Starr and Bales, [18])

At the time of the survey,19.1% of participants were smokers for a mean duration of 40 years (± 10.84). Current smokers consumed three to twenty packs of cigarettes weekly. Furthermore, 4.2% of the individuals drank three to seven glasses of alcohol per week. Diabetic participants represented 23.7%. Falls during the year past the survey occurred to 53.7%. Additionally, 62.2% of participants had HTN. Inner ear illness affected only 2.5% of individuals. Vitamin C was taken by 41%, and Vitamin A by 23%. Additionally, as shown in Tables 1 and 2 9.1% took anticoagulants.

**Table 2:** Special habits & chronic disease status of study participants:

	*N* (283)	%
Smoking Status		
-Non-smoker	144	50.9
-Ex smoker	85	30.0
-Current smoker	54	19.1
Current smokers (*n* = 54)		
Smoking Duration (in years)	40 ± 10.48 (10–60)	
Mean ± SD (Range)		
Cigarette smoking	54	100
Number of packs/weeks	7.48 ± 4.87 (3–20)	
Mean ± SD (Range)		
Others		
Opium	1	1.9
Cannabis	3	5.6
Alcoholic beverages (*n* = 12)	12	4.2
*Cups/week* ^a^		
Mean ± SD (Range)	5.70 ± 1.34 (3–7cups)	
Diabetes mellitus	67	23.7
Hypertension	176	62.2
Hypercholesterolemia	23	8.1
Inner Ear Disease	7	2.5
Falls during past year	152	53.7
Vit A	65	23
Vit C	116	41
Anticoagulant	54	19.1

^a^Cup = 150 ml

**Fig. 1:**
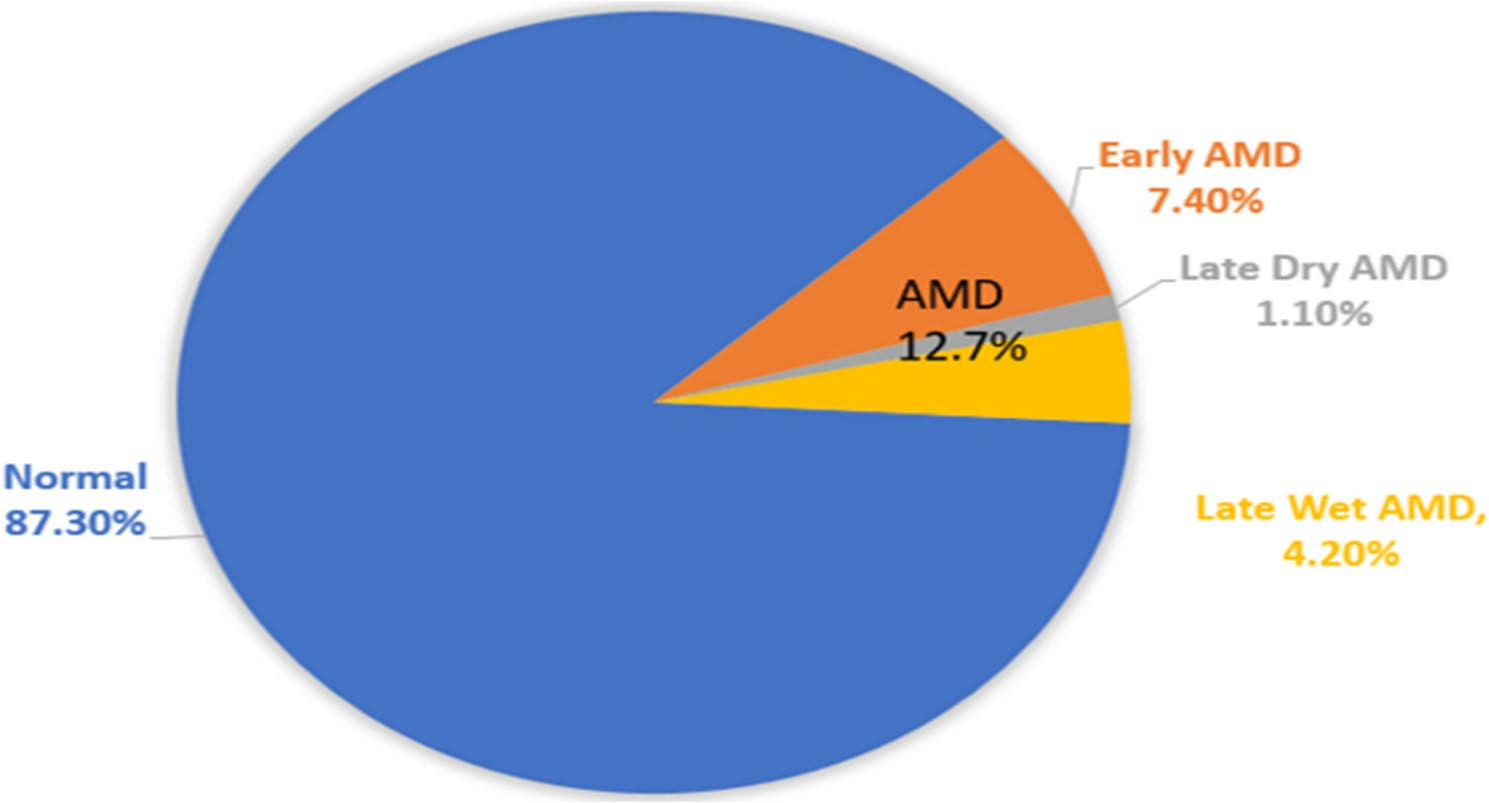
Prevalence of AMD and distribution of its types among study participants

**Fig. 2:**
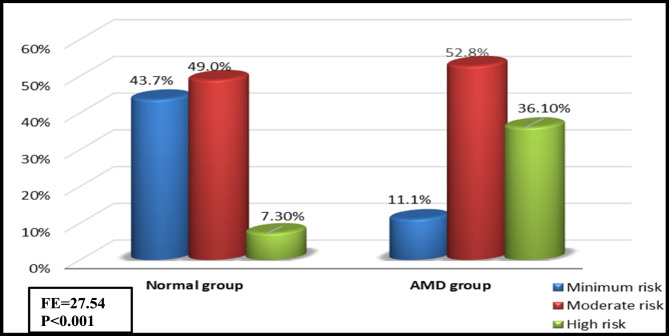
Comparison between normal and AMD participants regarding Morse Fall Scale categories

The results show an overall 12.7% prevalence rate of AMD in the studied sample. Early, late dry and late wet AMD were present in 58.3%, 8.3% and 33.3% of the participants, respectively, as shown in Figure 1. There is a significantly higher risk of falling among AMD group (p< 0.001) Fig. 2.

Female participants, elders with a prior family history of AMD, a lower level of education, and age of 75 years or older had a significantly higher prevalence of AMD than those in lower age groups. However, as indicated in Table 3, there was no statistically significant difference in BMI categories between the normal group and patients with AMD (*p* > 0.05).

**Table 3:** Relationship between characteristics of study participants & presence of AMD

	Normal group (*n* = 247)	AMD group (*n* = 36)	Test of significance	
	*N* (%)	*N* (%)	x^2^	*p*-value
Age groups^a^			19.97	< 0.001
60–64	12 (4.8)	1 (2.8)		
65–69	82 (33.2)	3 (8.3)		
70–74	74 (30)	7 (19.4)		
≥ 75	79 (32)	25 (69.5)		
Gender				
-Male	124 (50.2)	10 (27.8)	6.338	0.012
-Female	123 (49.8)	26 (72.2)		
Educational level			11.614	0.001
Below high school	79 (32)	22 (61.1)		
≤High school	168 (68)	14 (38.9)		
Previous occupation			2.442	0.295
-Blue Code	105 (42.5)	11 (30.6)		
-White Code	62 (25.1)	9 (25)		
-Pink Code	80 (32.4)	16 (44.4)		
Family history of AMD			FE^b^ 35.43	< 0.001
-Yes	2 (0.8)	7 (19.4)		
-No	245 (99.2)	29 (80.6)		
BMI Categories				
Under weight (< 23)	125 (50.6)	12 (33.3)		
Normal weight (23–30)	105 (42.5)	20 (55.6)	3.904	0.142
Overweight(> 31)	17 (6.9)	4 (11.1)		

^a^Fadel et al. (2016); ^b^*FE* Fisher Exact

It was found that smokers have a higher prevalence of AMD. The percentage of AMD was lower in participants who took vitamin A, vitamin C, and anticoagulants than in those who did not. Additionally, as seen in Table 4, AMD seems to be more common in people with DM and HTN.

**Table 4:** Comparison between normal group and AMD group regarding special habits & chronic disease status

	Normal group (*n* = 247)	AMD group (*n* = 36)	Test of significance	
	No (%)	No (%)	x^2^	*p*-value
Smoking^a^			23.922	< 0.001
Non-smoker	128 (51.8%)	16 (44.4%)		
Current smoker	37 (15.0%)	17 (47.2%)		
Past smoker	82 (33.2%)	3 (8.3%)		
Alcoholic beverages			FE^b^	0.052
Yes	8 (3.2%)	4 (11.1%)	4.795	
No	239 (96.8%)	32 (88.9%)		
Vit A				
Yes	63 (25.5%)	2 (5.6%)	7.068	0.008
No	184 (74.5%)	34 (94.4%)		
Vit C				
Yes	112 (45.3%)	4 (11.1%)	15.223	< 0.001
No	135 (54.7%)	32 (88.9%)		
Anticoagulants			7.747	
Yes	41 (16.6%)	13 (36.1%)		0.005
No	206 (83.4%)	23 (63.9%)		
DM				
Yes	41 (16.6%)	26 (72.2%)	53.798	< 0.001
No	206 (83.4%)	10 (27.8%)		
HTN				
Yes	143 (57.9%)	33 (91.7%)	15.24	< 0.001
No	104 (42.1%)	3 (8.3%)		
Hypercholesterolemia			FE^b^	
Yes	19 (7.7%)	4 (11.1%)	0.016	0.511
No	228 (92.3%)	32 (88.9%)		
Inner Ear Disease			FE^b^	1
Yes	6 (2.4%)	1 (2.8%)	0.492	
No	241 (97.6%)	35 (97.2%)		

In terms of how AMD affected functional limitations, there was a statistically significant association between AMD and a higher risk of falls (*p* < 0.001), with 52.8% and 36.1% of AMD patients having moderate and high risk of falls on the MFS, respectively, compared to 49% and 7.3% of normal participants. Furthermore, ADL and IADL scores showed a *p*-value of < 0.001, indicating that AMD was more closely linked to functional impairments. There is a statistically significant relationship between AMD and a higher frequency of falls (*p*-Value = 0.006), as 75% of AMD patients reported falling during the past year, compared to 50.6% in the normal group (Table 5).

**Table 5:** Comparison between normal and AMD groups regarding history of falls and functional limitations:

		Normal group (*n* = 247)	AMD group (*n* = 36)	Test of significance	
		No (%)	No (%)	Value	*p*-value
Mores Fall scale				FE 27.53	< 0.001
Minimum risk		108 (43.7%)	4 (11.1%)		
Moderate risk		121 (49.0%)	19 (52.8%)		
High risk		18 (7.3%)	13 (36.1%)		
ADL				t=−8.041	< 0.001
Mean ± SD		5.37 ± 1.436	3.33 ± 1.287		
IADL	Males	4.44 ± 1.26	2.2 ± 1.62	t = 5.28	< 0.001
	Females	7.07 ± 2.03	3.77 ± 2.03	t = 7.55	< 0.001
Falls history during the past year				*x*^2^ = 7.52	0.006
Yes		125 (50.6%)	27 (75%)		
No		122 (49.4%)	9 (25%)		

Table 6 displays an investigation of the factors linked to AMD using binary logistic regression. Advancing age, being female, ever-smoker, being diabetic increase the risk of AMD. Increasing duration of exposure to sun is significantly protective from AMD (*p*-Value = 0.027).

**Table 6:** Multivariate binary logistic regression analysis of factors associated with AMD:

	B (Coefficient)	Standard error	*P* value	Odds ratio	95% C.I.for EXP(B)	
					Lower	Upper
Age	0.073	0.029	0.013	1.075	1.015	1.139
Gender (ref: male)	1.932	0.702	0.006	6.906	1.744	27.342
Education (ref: illiterate)	0.071	0.134	0.593	1.074	0.826	1.396
DM (ref: non-diabetic)	2.283	0.495	< 0.0001	9.811	3.717	25.896
Smoking (ref: non-smoker)	0.472	0.403	0.242	1.603	0.727	3.534
Duration of sun exposure/day	−0.460	0.208	0.027	0.632	0.420	0.949
Vitamin A	−2.066	1.059	0.051	0.127	0.016	1.010
Vitamin C	−0.862	0.759	0.256	0.422	0.095	1.868
Anticoagulants	0.273	0.518	0.598	1.314	0.476	3.629
Constant	−8.764	2.479	0.000	0.000		

^a^Cigarette smoking was found in 100% of current smokers

^b^*FE* Fisher Exact

## Discussion

The current study sought to determine the prevalence of AMD in senior citizens residing in East Cairo’s geriatric facilities. A diagnosis of AMD was made for 36 (12.7%) of the 283 individuals. This is comparable to the findings of the European Eye Epidemiology (E3) Consortium, which found that the prevalence of AMD in people aged 70 and older was 16.2%. According to Wong et al., the prevalence of AMD in Europe was 18.3% [19]. Additionally, Rudnicka et al. found that 12.2% of UK people aged 80 and above suffered from AMD [20].

However, in Saudi Arabia, the prevalence of AMD was approximately 8.9% [21]. The present study’s higher prevalence could be explained by the diverse age distribution of the participants and the recent rise in the ageing population. Moreover, Fadel et al. found that 6.6% of patients enrolled in Alexandria University Hospital’s outpatient clinic had AMD. The limitations of Fadel’s study, as they mentioned, were that there were relatively few people aged 80 and above and that they were unlikely to have fundus examinations. This may also account for the discrepancy from the current study. This could have led to a misleading decrease in the prevalence of AMD [22]. A greater global prevalence of AMD of 27.7% was observed by Li et al. [23]. A prevalence of 24.8% for undiagnosed AMD, in Alabama was also reported [24].

An early diagnosis of AMD was made for 7.4% of the total participants in this study. It is comparable to the 6.8% estimate for early AMD in the Asian population, aged 40–79, as reported by Kawasaki et al., even though the prevalence for the same age range in the white population was slightly higher (8.8%) [25]. In Europeans, 11.2% of people had early AMD [19]. However, Fadel et al. claim that early AMD was just 5.3% [22]. The results of the current investigation showed that the prevalence of late AMD was 5.3%. It is higher than the prevalence reported in a number of previous studies, including the Blue Mountains Eye Study, which found late AMD to be 2.06%, and the Thessaloniki Eye Study, which found it to be 2.4% [22]. The inconsistent results could be explained by their use of various AMD classification methods, such as the Beckman or Rotterdam Classification [23].

The current study found that participants 75 years of age and above had the highest prevalence of AMD, indicating that growing older was a substantial risk factor for AMD. Other studies support this by demonstrating that the incidence of AMD was higher in people over 75 than in those under that age [26]. Fadel et al. also found that there were more AMD patients over 75 than AMD-free patients [22]. Additionally, the risk increased from 2% in the 50–59 age group to about 30% in the 75 + age group [27].

Women were more likely to have the disease (72.2%) than men (27.8%). The Beaver Dam Eye Study, the Blue Mountain Eye Study, and the Rotterdam Study of the Elderly are the three main studies that support this. A meta-analysis on the Europeans also found a connection between AMD and being a female [28]. According to Bourne et al., women were more likely than males to become blind from AMD [29]. This outcome is also consistent with the Age-Related Macular Degeneration Epidemiology Forecast, which linked the higher prevalence of the condition in females to their longer life expectancy [30]—a situation that is also true for the Egyptian population [31]. Choudhury et al. and other studies, however, found that Latin men were twice as likely to get AMD [32]. Additionally, research on Asians has shown that men are more likely than women to have AMD [33]. These results were explained by inclusion of large number of men in the higher age groups.

In this study, inadequate education was assumed to be linked to a higher percentage of AMD patients. This is in line with certain population-based research that found that those who taught high school or above had a lower risk of AMD [6, 34]. Since education was not statistically significant in the current study’s logistic regression, it is most likely not a direct risk but rather associated with other risky behaviors like smoking and poor nutrition [35].

According to the current study, smokers had a statistically significant higher prevalence of AMD than nonsmokers. This is in line with [36], who found that smoking was linked to AMD and that the presence of big drusen increased with pack years. Smokers had twice the chance of developing AMD, according to the US Twin Study of AMD [37]. In Korea, Moon et al. found a significant association between smoking and AMD (OR: 1.95) [38]. Further, Velilla et al. discovered that more than 10 pack-years increase the risk of AMD [39].

Similar to the findings of [40], there was a significant association between HTN and AMD in the current research. According to Goldacre et al. and the National Eye Institute (NEI), patients with hypertension had a twofold increased risk of AMD [41]. Additionally, hypertensive people are three times more likely to develop AMD, according to the Beaver Dam Eye Study (BDES). Xu et al. showed that compared to people receiving treatment for hypertension, those with long-term, poorly managed hypertension have a higher chance of developing AMD [42]. The development of AMD in hypertensives can be explained by at least *two different mechanisms*: humoral factors and hemodynamic damage. According to the first, oxidative stress brought on by capillary stretching from high blood pressure or flow turbulence in the arteries, as well as insufficient choroidal blood flow, may be factors in the formation of Choroidal Neovascularization (CNV) [43]. Furthermore, angiotensin II (Ang II), a hormone that is increased in hypertension, appears to be connected to AMD. Ang II causes tissue damage, inflammation, and endothelial dysfunction in the retina [44].

In the current study, there was an association between DM and AMD. According to the Blue Mountains and Barbados studies, diabetics had a 2.7-fold increased risk of AMD, which supports this [45]. According to Chen et al.‘s meta-analysis, DM is one of the numerous risk factors for AMD, particularly for late-stage AMD as opposed to early-stage AMD [46]. According to Vassilev et al., type II diabetics were more likely to develop AMD (OR, 1.38) [47]. He et al. from Taiwan found that throughout the course of a 15-year follow-up period, patients with diabetes had a higher overall annual incidence of AMD than those without diabetes [48]. It is yet unknown how diabetes and AMD are biologically related. Because hyperglycemia affects the Bruch membrane’s structure and function, the choroidal circulation, and the retinal epithelium, it probably raises the risk of AMD [45]. Other research, however, was unable to relate diabetes to AMD [22, 46]. As stated in the study’s limitations, the findings could be explained by the characteristics of non-respondents in comparison to respondents.

In the current study, anticoagulant use was significantly protective from AMD in the bivariate analysis. This aligns with the Age-Related Eye Disease Study’s recommendation that anti-inflammatory drugs, particularly aspirin, protect against AMD. Additionally, Christen et al. found that using low-dose aspirin every other day decreased the risk of AMD by a nonsignificant 18% after a 10-year follow-up when compared to a placebo [49]. Xu et al. found that taking low-dose aspirin over a 10-year period, particularly in elderly populations, reduced the risk of AMD by 20% [50]. In contrast, the Beaver Dam Eye Study and a few other studies validated the European survey’s finding that regular aspirin use increased the incidence of AMD by an odds ratio of 2.22 [51, 52]. These different results might be explained by censoring due to high dropout rates in these cohort studies [53].

According to the current study’s bivariate analysis, consuming vitamins, such as vitamin C and vitamin A, significantly reduces the risk of developing AMD. This is comparable to other studies on the impact of vitamins on AMD. These studies found that a high intake of carotenoids, vitamins C, A, and E, zinc, and omega-3 fatty acids was associated with a decrease in the incidence or progression of the illness [54, 55].

According to the current study, 53.7% of study participants experienced falls during the previous 12 months. It is comparable to the findings of Wood et al., who found that 54% of people had fallen at least once in the previous 12 months [56]. Ehrlich et al. showed that individuals with visual impairment had a higher probability of falling than those with intact vision and that 47% of elderly women reported falls during a 12-month follow-up [57]. A statistical association between AMD and a higher incidence of falls was discovered. According to Hong et al. and Marmamula et al., among residents in Indian geriatric homes, those with AMD experienced higher falls (38.0%; CI 32.2–44.0) than those without AMD (25.9%; 95% CI 22.8–29.1) [58, 59]. Furthermore, AMD patients were twice as likely to fall (16% vs. 8%) as controls, according to Szabo et al. [60].

Furthermore, the current study used the Morse Fall Scale to evaluate the impact of AMD on fall risk, which had not, as far as we know, been used in earlier research of AMD impact on patients. According to the Morse Fall Scale, 52.8% of AMD participants had a moderate risk of falling, and 36.1% had a high risk. This strongly implies a connection between AMD and an increased risk of falls.

The impact of AMD on ADL and IADL as a reflection of older adults’ lives was also examined in the current study. When comparing AMD patients to their normal peers, it revealed a statistically significant decrease in ADL and IADL. Similarly, Gopinath et al. reported that AMD patients had a threefold increased risk of ADL impairment and a twofold increased risk of IADL disability [61]. Additionally, Szabo et al. found that AMD patients required four times as much assistance with daily tasks as controls (29% vs. 7%) [60]. A US clinic-based study showing a two-fold higher likelihood of IADL impairment among AMD patients further supports this [7].

### Limitations

The Self- reported data obtained from the interview questionnaire in this cross-sectional study, carries the possibility of recall bias. Some special habits like alcoholic beverage consumption couldn’t be investigated well due to cultural limitations.

## Conclusion and recommendations

According to the results of the current cross-sectional study, AMD is more common than previously reported in Egypt. The most prevalent type was early AMD, which was followed by late wet AMD. Age and gender were non-modifiable risk factors for AMD. Additionally, smoking, diabetes mellitus, and hypertension are important risk factors for AMD. Exposure to sun for longer duration seems to have a protective effect from AMD. Compared to older people without AMD, AMD patients are more likely to fall. The greatest way to reduce the risk of AMD seems to be through prevention. Since there is still disagreement on the available treatments. Given the high prevalence of AMD, there is a push to increase public awareness of the importance of routine eye examinations. Health education about risk factors like smoking, diabetes, and hypertension, as well as the benefits of vitamin supplements and longer sun exposure, is an essential preventive measure. To have a better understanding of AMD risk factors, longitudinal research with a larger sample size should be conducted.

## Supplementary Information

Supplementary Material 1.

## Data Availability

Data is Available on reasonable request.

## Abbreviations

ADL: Activities of daily living
AMD: Age Related Macular Degeneration
IADL: Instrumental activities of daily living
AREDS: Age-Related Eye Disease Study
BMI: Body Mass Index
MCI: Mild cognitive impairment
MMSE: Mini-mental State Examination
