# Supplementary material for: Epidemiology of age-related macular degeneration among elderly in geriatric homes, East Cairo, Egypt
Source: BMC Public Health. 2025 Jul 18;25:2495. doi: 10.1186/s12889-025-23680-6 (PMC12272965; doi:10.1186/s12889-025-23680-6)
Supplement: Supplementary file 1 — Supplementary Material 1. [file 12889_2025_23680_MOESM1_ESM.docx]

71 participants were excluded by examination due to having eye diseases causing significant optic media opacity or optic nerve affection according to the exclusion criteria.

41 participants were excluded by history of previous eye surgery: glaucoma, cataract or retinal surgeries.

11 participants were excluded due to mild cognitive impairment detected by mini mental state examination.

**Figure (S1): Sampling of geriatric homes sharing in study**

283 elderly persons were included in final results

354 elderly persons underwent eye examination.

395 elderly persons started the questionnaire.

406 elderly persons were interviewed
